# Supplementary figures and images for: Genomic survey maps differences in the molecular complement of vesicle formation machinery between Giardia intestinalis assemblages
Source: PLoS Negl Trop Dis. 2023 Dec 18;17(12):e0011837. doi: 10.1371/journal.pntd.0011837 (PMC10758263; doi:10.1371/journal.pntd.0011837)

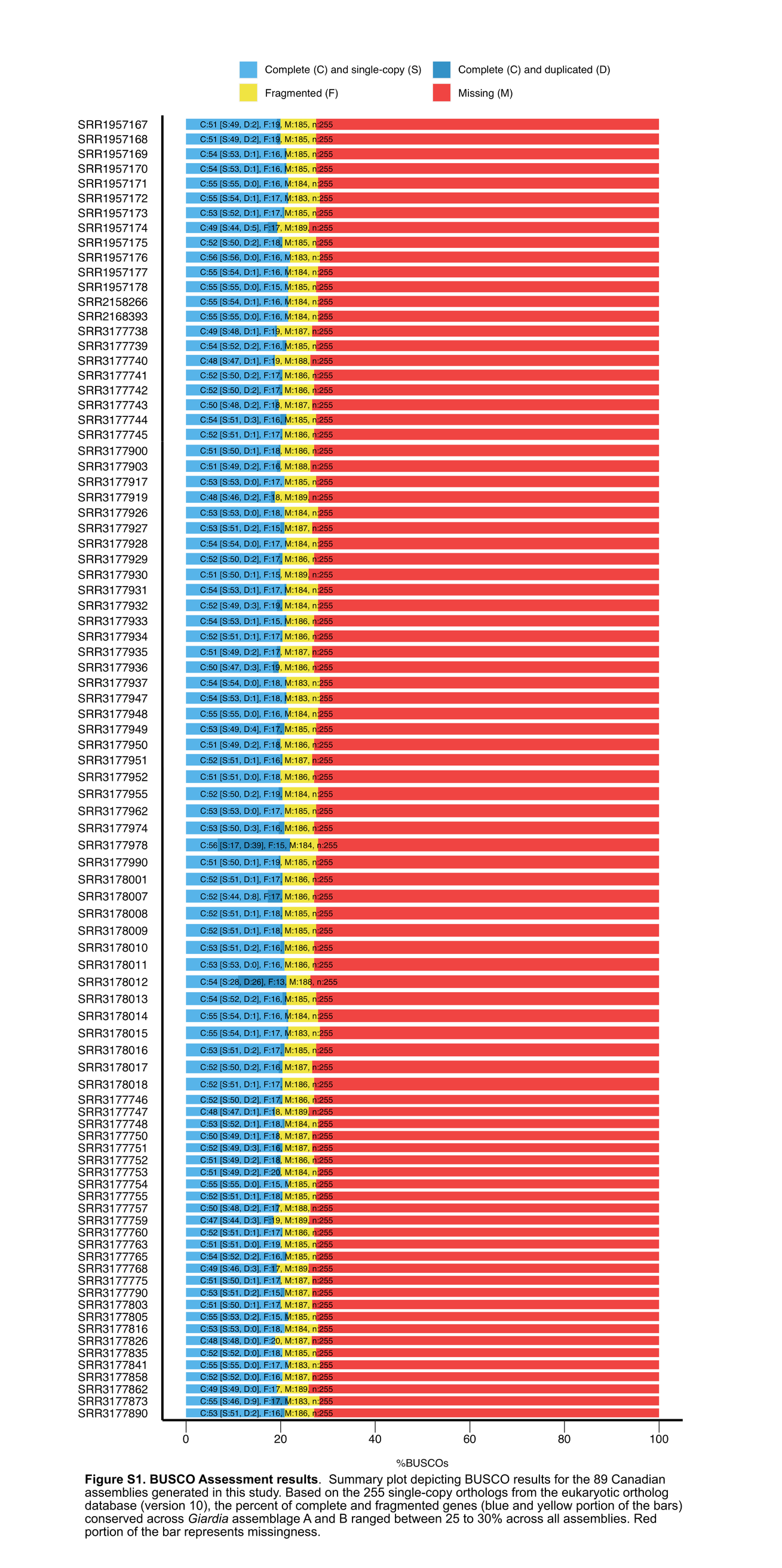

Supplement: S1 Fig — Summary plot depicting BUSCO results for the 89 Canadian assemblies generated in this study. Based on the 255 single-copy orthologs from the eukaryotic ortholog database [version 10], the percent of complete and fragmented genes [blue and yellow portions of the bars] conserved across Giardia assemblage A and B ranged from 25 to 30% across all assemblies. Red portion of the bar represents missingness. (TIF) [file pntd.0011837.s011.tif]
